# Exogenous proline mitigates toxic effects of cadmium via the decrease of cadmium accumulation and reestablishment of redox homeostasis in *Brassica juncea*

**DOI:** 10.1186/s12870-022-03538-4

**Published:** 2022-04-08

**Authors:** Yuanduo Wang, Piaopiao Tan, Liang Chang, Zheming Yue, Chaozhen Zeng, Mei Li, Zhixiang Liu, Xujie Dong, Mingli Yan

**Affiliations:** 1 Hunan Provincial Key Laboratory of Forestry Biotechnology, College of Life Science and Technology, Central South University of Forestry and Technology, 410004 Changsha, China; 2 International Cooperation Base of Science and Technology Innovation on Forest Resource Biotechnology of Hunan Province, Central South University of Forestry and Technology, 410004 Changsha, China; 3 Hunan Provincial Key Laboratory of Crop Germplasm Innovation and Utilization, Hunan Agricultural University, 410128 Changsha, China; 4 Crop Research Institute, Hunan Academy of Agricultural Sciences, 410125 Changsha, China; 5 Hunan Key Laboratory of Economic Crops Genetic Improvement and Integrated Utilization, Hunan University of Science and Technology, 411201 Xiangtan, China

**Keywords:** *Brassica juncea*, Cadmium tolerance, Reactive oxygen species, Oxidative stress, Antioxidant enzymes

## Abstract

**Background:**

As a vital osmoticum, proline has an important role in enhancing the tolerance of plants to environmental stress. It is unclear whether the application of exogenous proline can improve the tolerance of *Brassica juncea* to cadmium (Cd).

**Results:**

This study investigated the effects of different concentrations of proline (20, 40, 60, 80, and 100 mg/L) under Cd stress at different times (0 d, 2 d, and 7 d) on the growth and physiology of *B. juncea*. Treatment with exogenous proline not only increased the content of proline in *B. juncea* but also alleviated Cd-induced seedling growth inhibition via the maintenance of higher photosynthetic pigment content and cell viability and a decrease in the content of Cd. Moreover, it increased the activities of antioxidant enzymes and the glutathione/glutathione disulfide ratio to reduce the accumulation of reactive oxygen species. Compared with other concentrations, 60 mg/L of exogenous proline was the most effective at mitigating Cd toxicity in *B. juncea*.

**Conclusions:**

Exogenous proline treatment enhanced the tolerance to Cd via a decrease in Cd accumulation and reestablishment of the redox homeostasis in *B. juncea.*

**Supplementary Information:**

The online version contains supplementary material available at 10.1186/s12870-022-03538-4.

## Background

Cadmium (Cd) is a toxic metal element, which is difficult to degrade [1]. It is one of the most aggressive and persistent heavy metals in the natural environment. The dangers of Cd include two aspects: (1) Cd reduces the yield by interfering with the lifecycle of the plant; and (2) Cd is easily absorbed and accumulated by plants, which enables it to enter the food chain and deleteriously affects animals and humans [2]. *The National Soil Pollution Status Survey Bulletin* shows that the rate of Cd in China exceeded 7.0%, ranking it first among inorganic pollutants [3]. Cd can hinder plant growth, inhibit chlorophyll synthesis, and decrease plant biomass, gas exchange attributes, nitrogen utilization, and the activities of assimilation enzymes, as well as interfere with the antioxidant systems [4–7].

A burst of reactive oxygen species (ROS) is induced in plants under abiotic stresses, thereby hindering their normal growth and development [8, 9]. Plants protect the cell mechanisms against oxidative stress by accumulating osmotic compounds. Proline is the most common osmolyte, which stabilizes the osmotic difference between the extracellular environment and the cytoplasm [10]. In addition, it can protect plant cells from oxidative stress by inhibiting the production of ROS [11]. The accumulation of proline is regarded as an adaptive strategy in response to heavy metal stress in plants [12, 13]. It has been reported that treatment with proline significantly reduced the loss of photosynthetic pigments and counteracted mercury (Hg)-triggered oxidative stress in coriander (*Coriandrum sativum* L.). seedlings [14]. Proline can reduce the lipid peroxidation of rice (*Oryza sativa*) seedlings under Cr (VI) stress, which manifests as a decrease in the content of malondialdehyde (MDA) and root cell activity [15]. The foliar spraying of proline significantly improved the growth, net photosynthetic rate (Pn), the content of chlorophyll, leaf carbonic anhydrase activity, and quantum yield of photosystem II, as well as the activities of antioxidant enzymes in *Brassica juncea* [16]. Exogenous proline alleviated the adverse effects of Cd on date palm (*Phoenix dactylifera* L.) and olive (*Olea europaea* L. cv. Chemlali). It reduced the oxidative damage caused by Cd accumulation, established a better level of plant growth, water status, and photosynthetic activity, and resulted in higher activities of antioxidant enzymes in the roots and leaves [17, 18].

*B. juncea* has been identified as a metal accumulator that is ecologically and economically important, and it is a large biomass crop plant within the Brassicaceae family [19]. Increasing amounts of evidence suggest that it has a remarkable capacity to take up a large number of Cd molecules from soils contaminated with Cd [20, 21]. However, whether treatment with exogenous proline alleviates the toxicity of Cd in *B. juncea* remains unknown. Thus, the aim of this study was to investigate the effects of exogenous proline on plant growth, biomass, contents of Cd and proline, cell viability, the activities of antioxidant enzymes, including superoxide dismutase (SOD), peroxidase (POD), ascorbate peroxidase (APX), catalase (CAT), and the contents of non-enzymatic products, including chlorophyll, glutathione (GSH), and GSH/glutathione disulfide (GSSG), by exploring the potential ameliorative efficacy of proline on Cd toxicity in *B. juncea*.

## Results

### Effects of exogenous proline on the endogenous proline content in seedlings

**Fig. 1:**
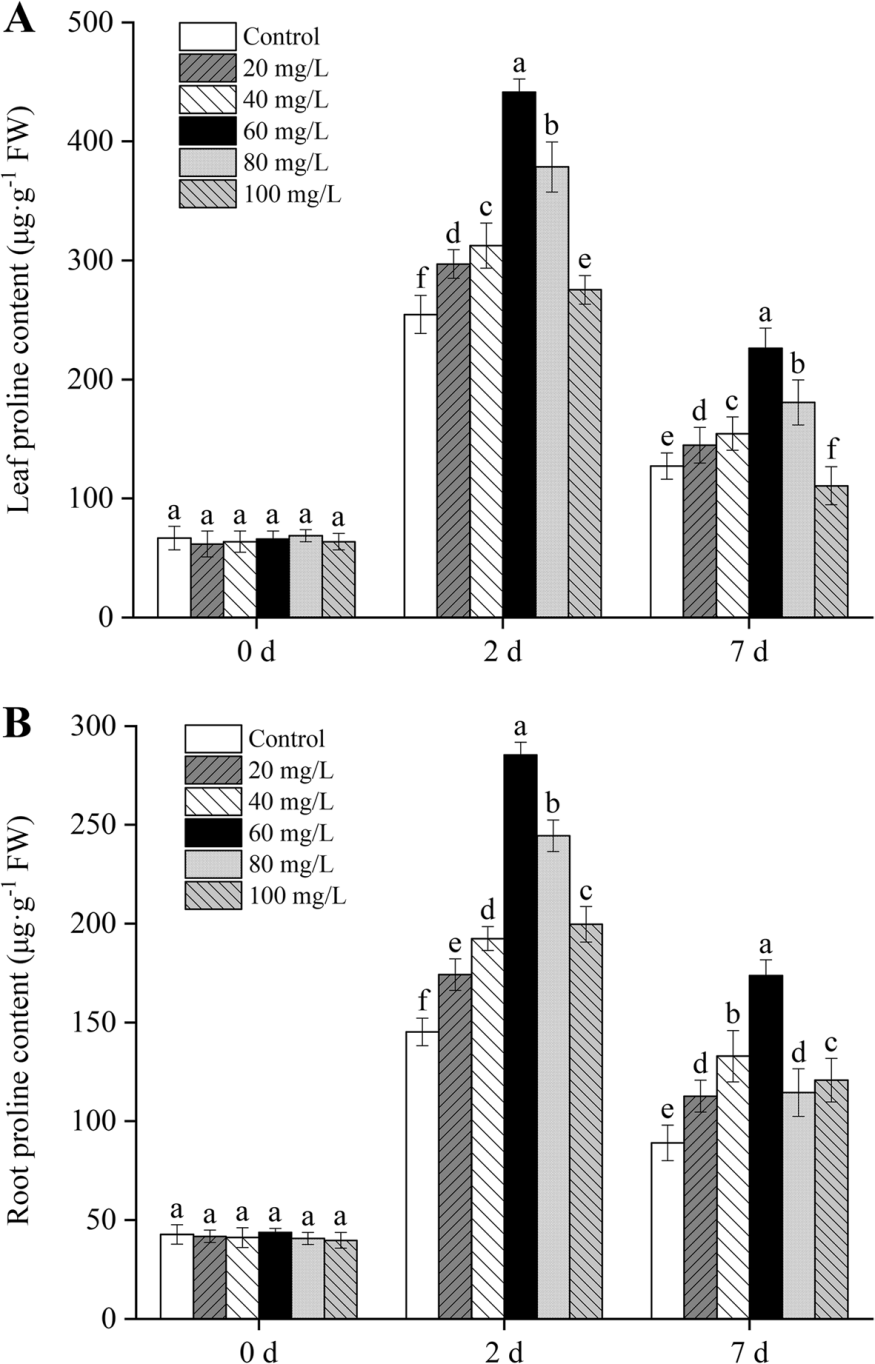
Effect of exogenous proline on the proline content in leaves (**A**) and roots (**B**) of *Brassica juncea* seedlings under Cd stress. All the analyses were performed with three replicates. Error bars represent the standard deviations (SD). The different letters in the group indicate significantly different values between treatments (*P* < 0.05)

The application of exogenous proline significantly increased (*P* < 0.05) the content of endogenous proline in *B. juncea* plants stressed with Cd (Fig. 1). At an exogenous proline concentration of 60 mg/L, the increase in endogenous proline content was the most significant in *B. juncea* leaves and roots. The plants were subjected to Cd stress for 2 d and 7 d, and the concentration of endogenous proline increased 2.8- and 2.4-fold in the leaves, whereas it increased 4.5- and 3.7- fold in the roots compared with the control group, respectively.

### Effect of exogenous proline on plant growth

**Fig. 2:**
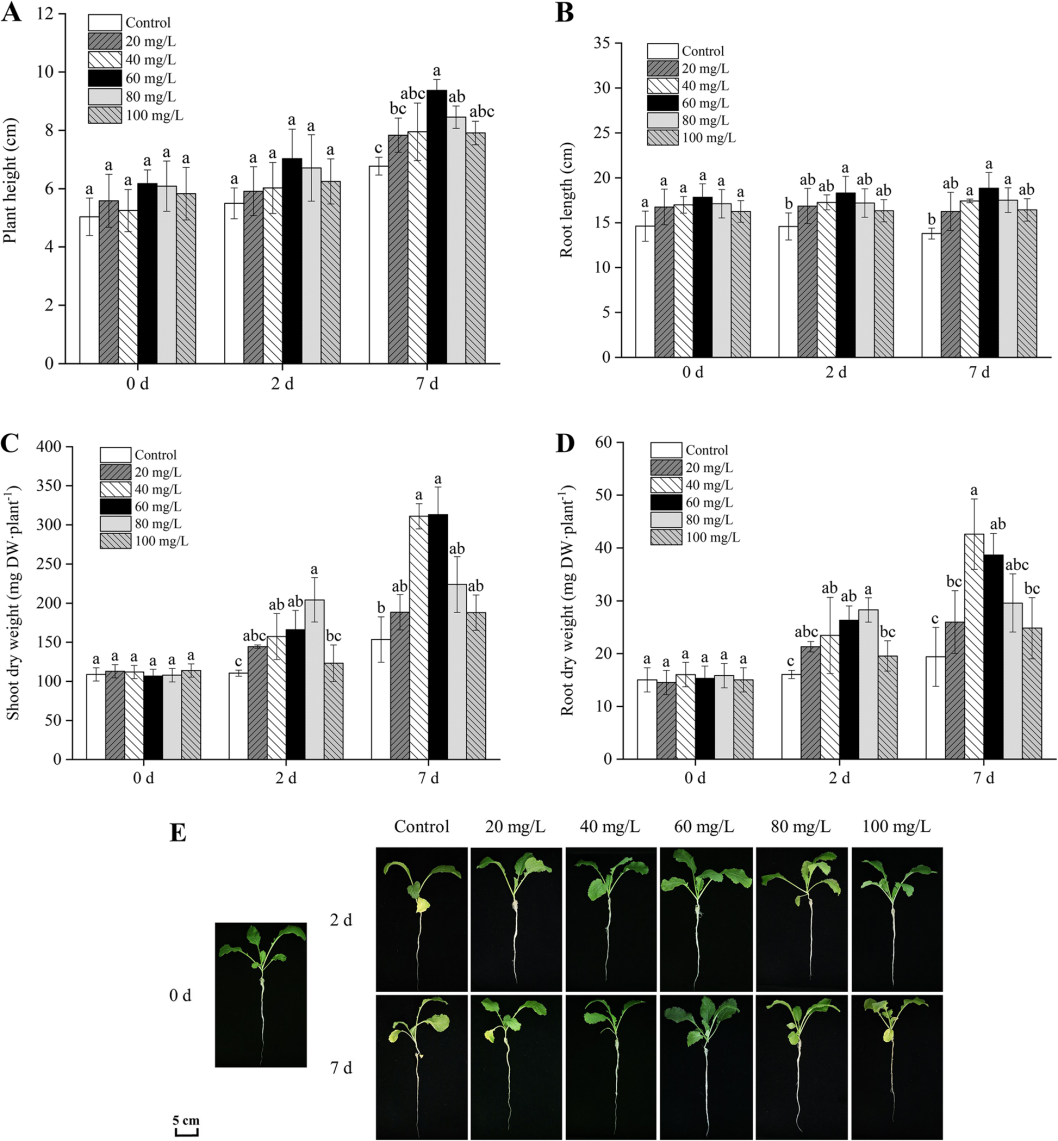
Effect of exogenous proline on the plant height (**A**), root length (**B**), shoot dry weight (**C**), root dry weight (**D**), and plants (**E**) of *Brassica juncea* under Cd stress. All the analyses were performed with three replicates. Error bars represent the standard deviations (SD). The different letters in the group indicate significantly different values between treatments (*P* < 0.05)

As shown in Fig. 2 A, B, and E, supplementation with exogenous proline mitigated the inhibitory effect of Cd stress at different times on the plant height and root length. The plants treated with 60 and 80 mg/L proline grew remarkably higher (*P* < 0.05) compared with those in the control group following Cd treatment for 7 days, whereas the root length of plants treated with 40, 60, and 80 mg/L proline increased significantly (*P* < 0.05). The 60 mg/L treatment had the most obvious effect on the growth of Cd-stressed *B. juncea* seedlings, which increased by 38.4% and 36.79% in plant height and root length, respectively, compared with the control group.

Figure 2 C and D show that treatment with exogenous proline ameliorated the loss of shoot and root dry weight under Cd stress at 2 d and 7 d, respectively. In comparison with the control plants, the treatment of plants that had been subjected to 2 d of Cd stress with exogenous proline (40, 60, and 80 mg/L) significantly alleviated the reduction in shoot dry weight (*P* < 0.05). After Cd treatment for 7 days, the shoot and the root dry weights of *B. juncea* seedlings were the most clearly affected after 40 mg/L and 60 mg/L proline treatment, respectively (*P* < 0.05). Compared with the control group, the shoot and root dry weight increased by 46.69% and 119.58%, respectively. Exogenous proline significantly increased the plant height, root length, and shoot and root dry weight, indicating that it alleviated the stress of Cd on the growth of *B. juncea* seedlings.

As shown in Fig. S1, the plant height and dry weight of the seedlings treated solely with 60 mg/L proline for 7 days were higher than those of the control. This illustrates that 60 mg/L proline is also beneficial for the normal growth of *B. juncea*.

### Effects of exogenous proline on chlorophyll and carotenoid contents, net photosynthetic rate, stomatal conductance, intercellular carbon dioxide concentration, and transpiration rate

**Fig. 3:**
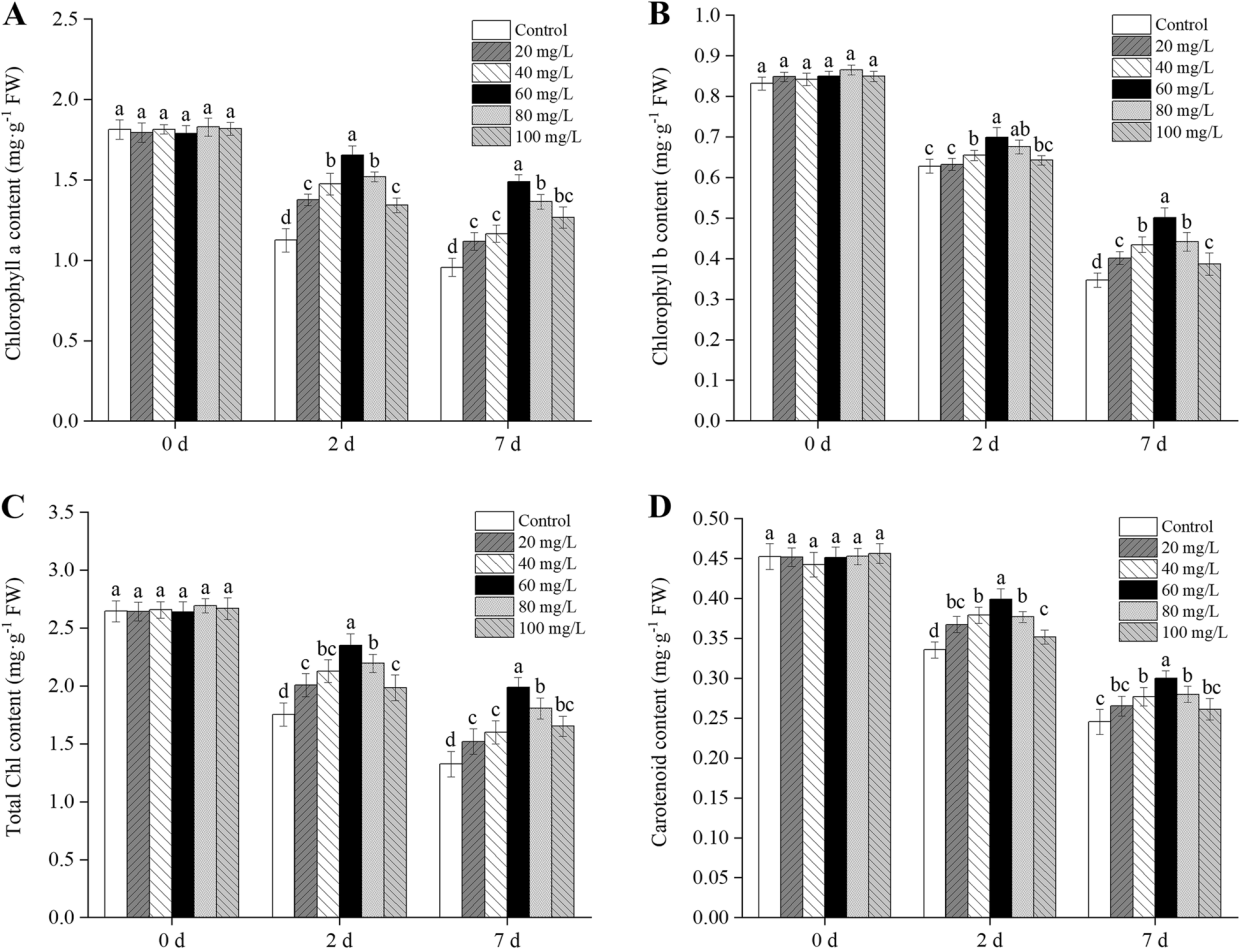
Effect of exogenous proline on the chlorophyll a content (**A**), chlorophyll b content (**B**), total chlorophyll content (**C**), and carotenoid content (**D**) of *Brassica juncea* seedlings under Cd stress. All the analyses were performed with three replicates. Error bars represent the standard deviations (SD). The different letters in the group indicate significantly different values between treatments (*P* < 0.05)

As shown in Fig. 3, with the increase of exogenous proline, similar trends were found in the contents of chlorophyll a, chlorophyll b, total chlorophyll, and carotenoids in Cd-induced *B. juncea* leaves for 2 d and 7 d, showing a trend of first increasing and then decreasing. These results indicated that 60 mg/L of exogenous proline was most effective at maintaining the contents of photosynthetic pigments in *B. juncea* leaves under 2 d and 7 d of Cd stress (*P* < 0.05). Compared with the control group, the contents of chlorophyll a, chlorophyll b, total chlorophyll, and carotenoids following treatment with 60 mg/L of exogenous proline increased by 46.77%, 11.26%, 34.06%, and 18.98% after 2 d of treatment with Cd, respectively, whereas it increased by 55.44%, 44.49%, 50.04% and 22.17% after 7 d of treatment with Cd, respectively.

**Fig. 4:**
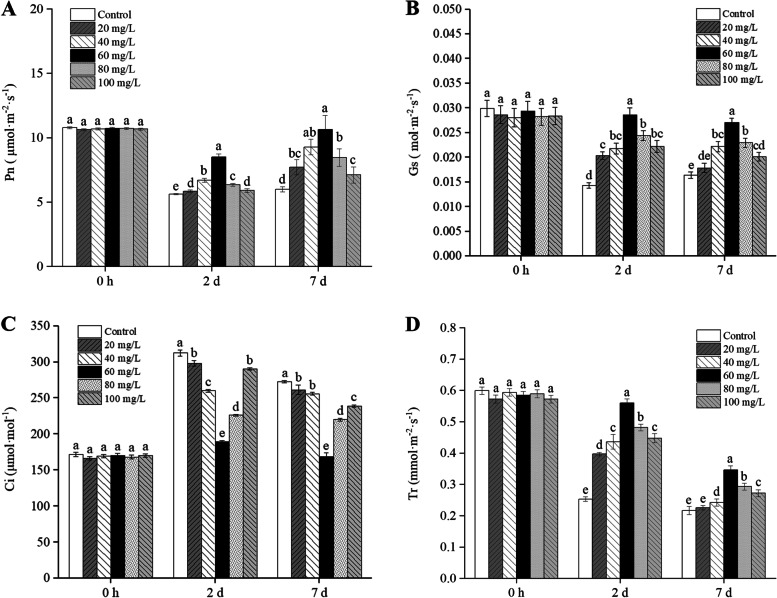
Effect of exogenous proline on the net photosynthetic rate (Pn) (**A**), stomatal conductance (Gs) (**B**), intercellular carbon dioxide concentration (Ci) (**C**) and transpiration rate (Tr) (**D**) of *Brassica juncea* seedlings under Cd stress. All the analyses were performed with three replicates. Error bars represent the standard deviations (SD). The different letters in the group indicate significantly different values between treatments (*P* < 0.05)

As shown in Fig. 4, the addition of exogenous proline significantly increased the Pn of *B. juncea* leaves with the increase in Cd treatment time (*P* < 0.05) and reached a maximum at a concentration of 60 mg/L. The stomatal conductance (Gs), intercellular carbon dioxide concentration (Ci), and transpiration rate (Tr) of *B. juncea* decreased with increasing Cd treatment times, but at a concentration of 60 mg/L, the Gs and Tr were significantly higher than those of the control, and the Ci was significantly lower than that of the control (*P* < 0.05).

### Effect of exogenous proline on cell viability in seedlings

**Fig. 5:**
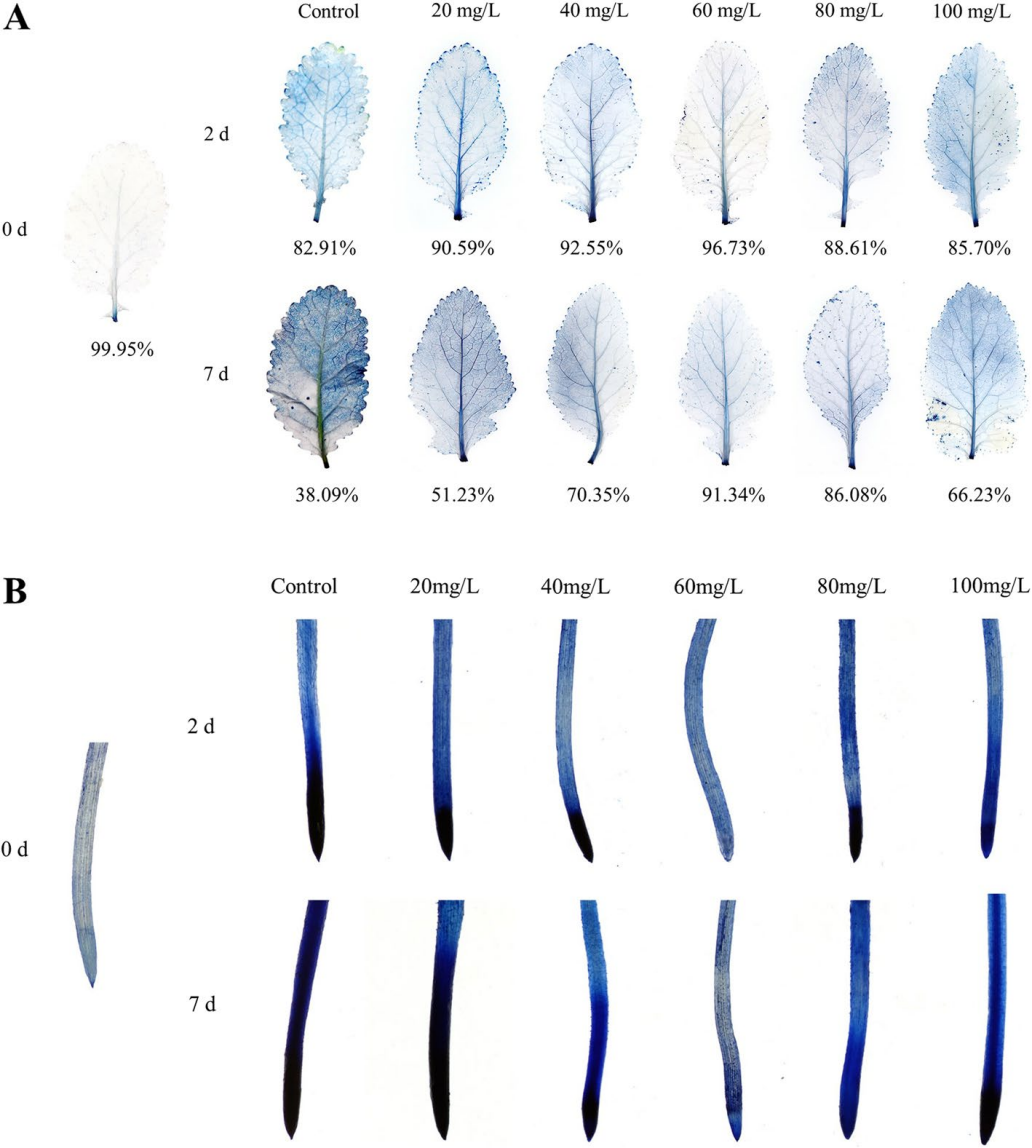
Results of Evans blue staining on leaves (**A**) and root tips (**B**) of *Brassica juncea* under Cd stress

Figure 5 A shows that the relative cell viability of *B. juncea* leaves treated with exogenous proline was significantly higher than that of the control. After Cd treatment for 2 d and 7 d, the relative cell viability of *B. juncea* leaves reached its maximum when the proline concentration was 60 mg/L, which were 96.73% and 91.34%, respectively. The results indicate that adding an appropriate amount of proline is favorable to the relative cell viability of *B. juncea* under Cd stress.

As shown in Fig. 5B, as the concentration of exogenous proline increases, the root tip changes from dark to bright, and then dark. When the concentration of exogenous proline was 60 mg/L, the relative cell viability reached its maximum.

### Effect of exogenous proline on Cd content in seedlings

**Fig. 6:**
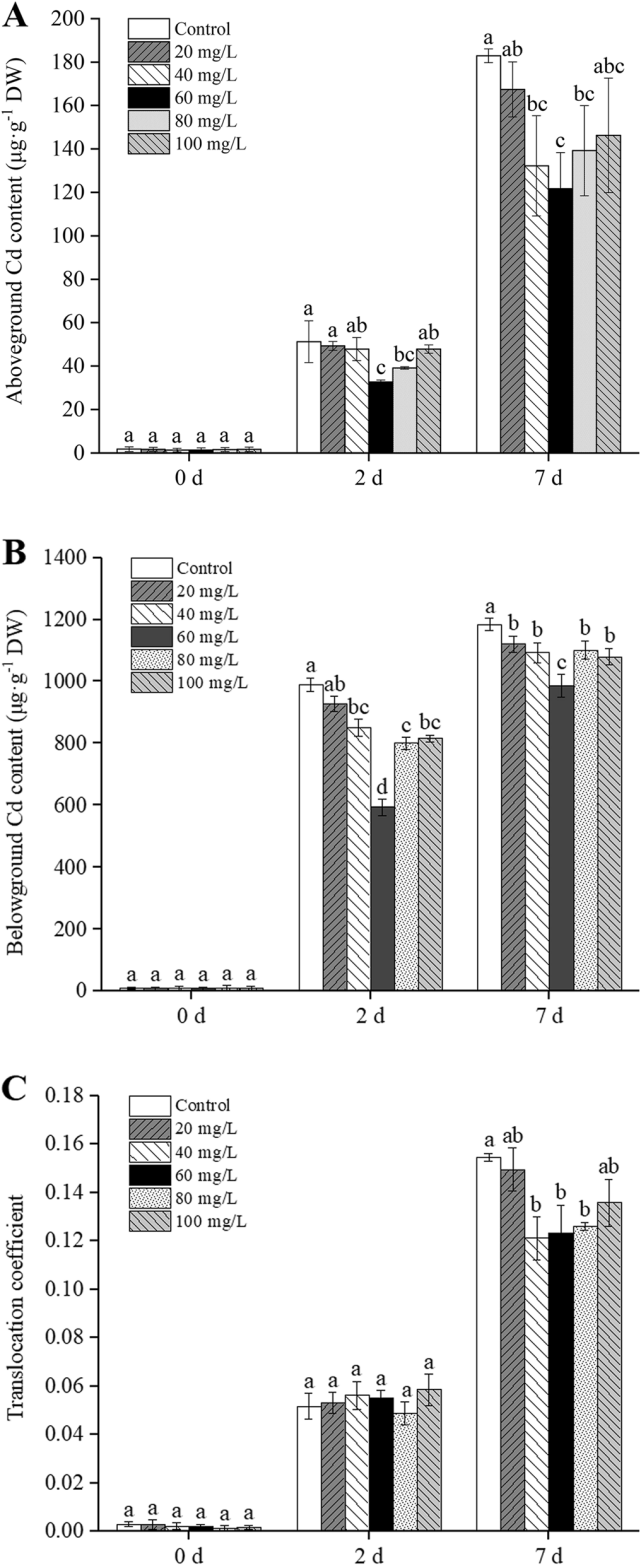
Effect of exogenous proline on the aboveground Cd content (**A**), belowground Cd content (**B**), and translocation coefficients (**C**) of *Brassica juncea* seedlings under Cd stress. All the analyses were performed with three replicates. Error bars represent the standard deviations (SD). The different letters in the group indicate significantly different values between treatments (*P* < 0.05)

As shown in Fig. 6, the Cd content of *B. juncea* seedlings increased significantly with the increase in time of treatment with Cd. After Cd stress for 7 days, the belowground contents of Cd in the *B. juncea* seedlings treated with different concentrations of exogenous proline decreased significantly compared with the control (*P* < 0.05), whereas the aboveground contents decreased significantly in treatments with 40, 60, and 80 mg/L proline. Among them, treatment with 60 mg/L proline treatment reached the lowest value, a decrease of 33.47% and 16.72% compared with those of the control group, respectively. No significant differences in translocation coefficients (the ratio of the element’s presence in the plant’s aboveground parts compared to that in the plant’s belowground parts) were observed in the treatments following Cd stress for 2 d. Following Cd stress for 7 days, exogenous proline (40, 60, and 80 mg/L) significantly decreased the translocation coefficients compared with the control (*P* < 0.05).

### Histochemical staining of ROS

**Fig. 7:**
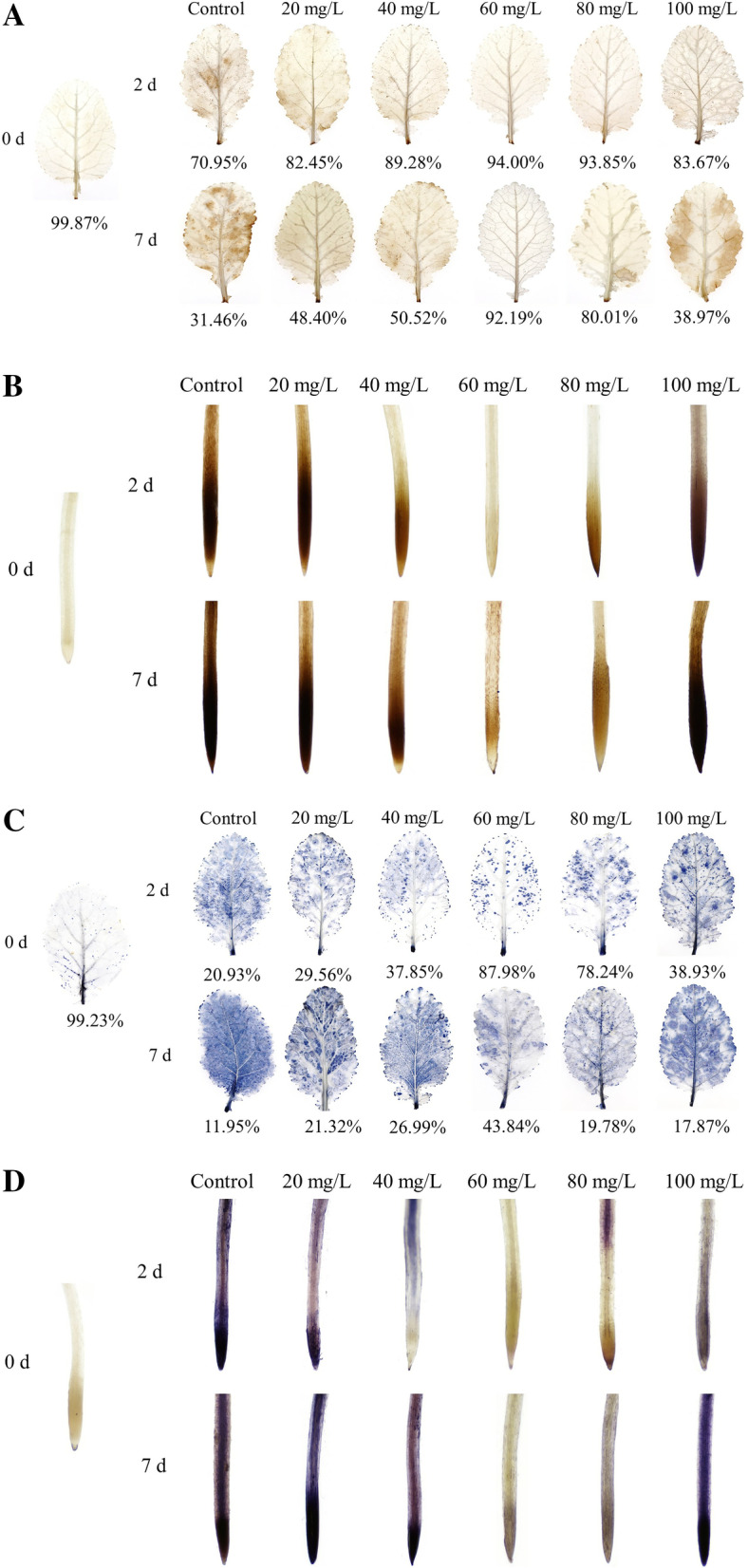
DAB staining results of leaves (**A**) and roots (**B**), NBT staining results of leaves (**C**) and roots (**D**) of *Brassica juncea* under Cd stress. DAB, 3,3′-diaminobenzidine; NBT, nitro blue tetrazolium

The presence of dark blue and dark brown spots indicates serious oxidative rupture under heavy metal stress [22]. As shown in Fig. 7, the leaves and roots of *B. juncea* stained with 3,3′-diaminobenzidine (DAB) and nitro blue tetrazolium (NBT) became deeper with the increase in Cd stress time, indicating that the oxidative rupture became increasingly serious. Following exogenous treatment with proline, the color of Cd-stressed *B. juncea* leaves and roots by NBT and DAB staining were significantly lighter than those of the control. Exogenous proline, at a concentration of 60 mg/L, was the most effective at mitigating Cd toxicity in the leaves and roots. Therefore, the accumulation of hydrogen peroxide (H_2_O_2_) and superoxide anions (O_2_^−^) decreased significantly after Cd-stressed *B. juncea* seedlings were treated with exogenous proline.

### Effects of exogenous proline on antioxidant enzyme activities and MDA content in seedlings

**Fig. 8:**
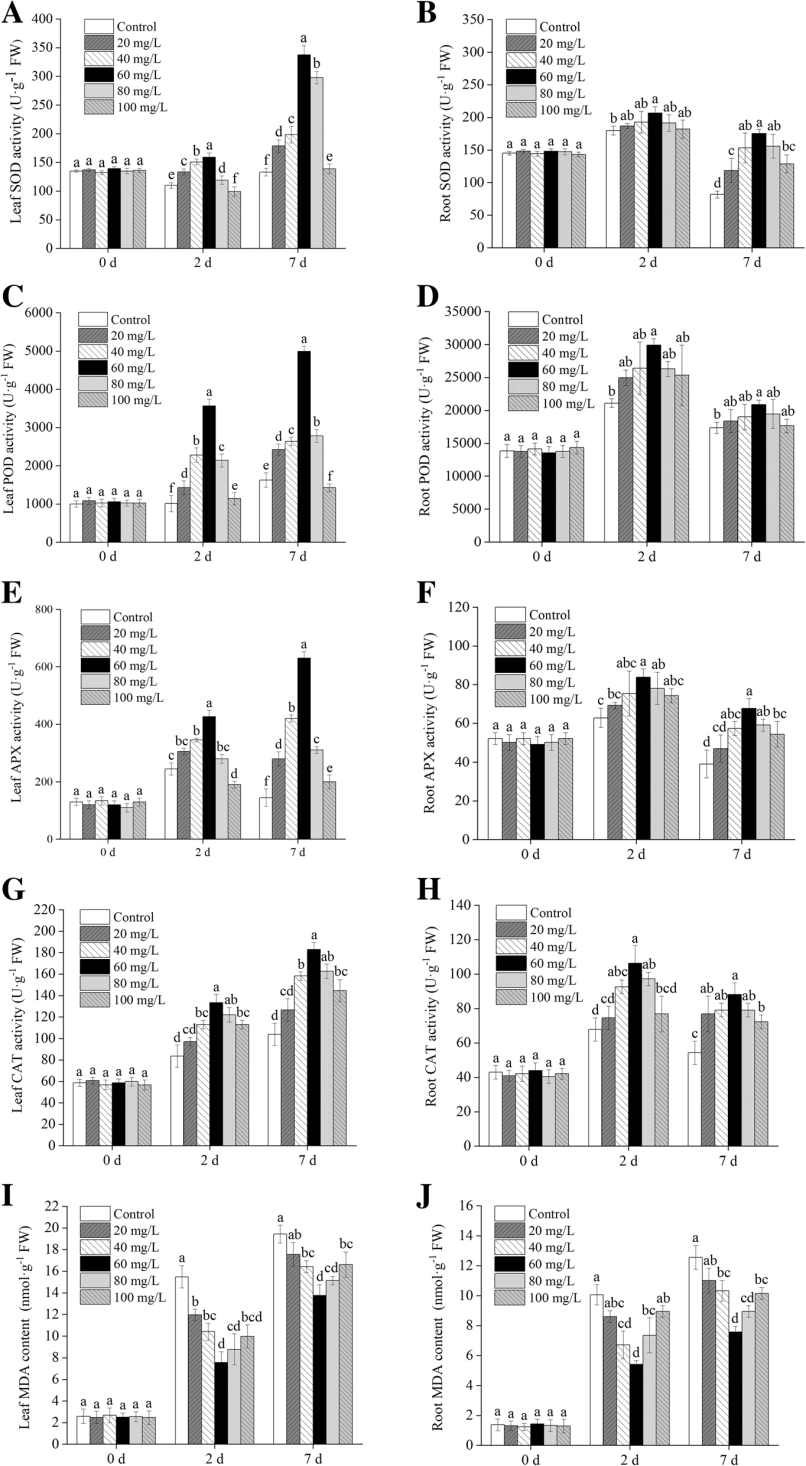
Effects of exogenous proline on antioxidation enzymes activities of SOD (**A**, **B**), POD (**C**, **D**), APX (**E**, **F**) and CAT (**G**, **H**) and the content of MDA (**I**, **J**) in the leaves and roots of *Brassica juncea* under Cd stress, respectively. All the analyses were performed with three replicates. Error bars represent the standard deviations (SD). The different letters in the group indicate significantly different values between treatments (*P* < 0.05). APX, ascorbate peroxidase; CAT, catalase; MDA, malondialdehyde; POD, peroxidase; SOD, superoxide dismutase

As illustrated in Fig. 8, under 2 d of Cd stress, compared with the control, treatment with 60 mg/L exogenous proline was the most effective at increasing the activities of SOD, POD, APX, and CAT in the leaves by 44.38%, 252.56%, 73.88%, and 59.52% respectively, whereas they increased by 14.88%, 41.75%, 33.53%, and 58.82% in the roots compared with the control group, respectively (*P* < 0.05). Following Cd treatment for 7 d, they increased by 153.75%, 206.97%, 334.48%, and 75.24% in the leaves, respectively, whereas they increased by 53.35%, 16.96%, 42.32%, and 63.64% in the roots, respectively. Therefore, the application of the appropriate amount of exogenous proline can enhance the activities of SOD, POD, APX, and CAT in *B. juncea* seedlings, which can effectively alleviate Cd stress. Consistent with the enhancement of activities of antioxidant enzymes, the content of MDA, a product of the peroxidation of membranes, was reduced.

### Effects of exogenous proline on the contents of reduced glutathione, oxidized glutathione, and phytochelatins and the activities of glutathione reductase in seedlings

**Fig. 9:**
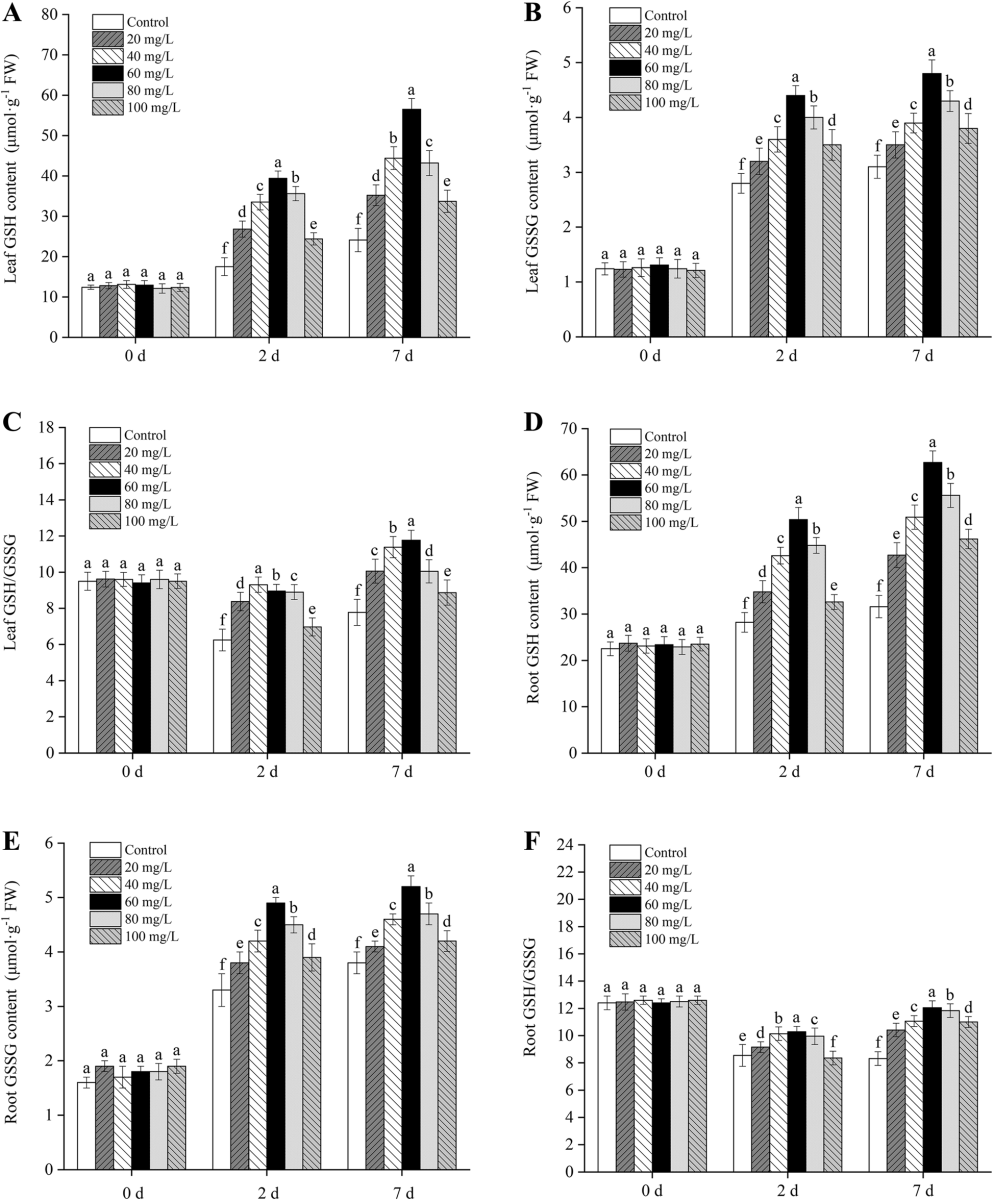
Effect of exogenous proline on the leaf GSH content (**A**), leaf GSSG content (**B**), leaf GSH/GSSG ratio (**C**), root GSH content (**D**), root GSSG content (**E**), and root GSH/GSSG ratio (**F**) of *Brassica juncea* seedlings under Cd stress. All the analyses were performed with three replicates. Error bars represent standard deviations (SD). The different letters in the group indicate significantly different values between treatments (*P* < 0.05). GSH, glutathione; GSH/GSSG, ratio of glutathione/glutathione disulfide

As shown in Fig. 9, with the extension of Cd treatment, the contents of GSH and GSSG in *B. juncea* leaves and roots increased, whereas the ratio of GSH/GSSG decreased and then increased. The GSH/GSSG ratio first increased and then decreased as the concentration of exogenous proline increased. Under different times of Cd stress, exogenous proline remarkably increased the contents of GSH and GSSG and the GSH/GSSG ratio in the *B. juncea* leaves and roots (*P* < 0.05), and reached its maximum value when the concentration of exogenous proline was 60 mg/L.

The application of exogenous proline did not affect the contents of phytochelatins (PCs) in plants that were not subjected to Cd stress (Fig. S2). Compared with the control group, Cd stress significantly increased the content of PCs in the leaves and roots, especially in the roots (*P* < 0.05). After 7 days of Cd stress, the contents of PCs in the roots were elevated by 79.39% and 120.79%, respectively. In addition, 60 mg/L proline treatment resulted in an increase in the contents of PCs in roots by 60.19% and 18.25%, respectively, compared with Cd stress alone.

As shown in Fig. S3, after 7 days of Cd treatment, compared with the control, the activities of glutathione reductase (GR) of the leaves and roots increased significantly by 21.49% and 47.97%, respectively (*P* < 0.05). Compared with Cd stress alone, the GR activities in the leaves and roots of *B. juncea* treated with Cd and 60 mg/L proline were significantly increased by 10.20% and 16.71%, respectively (*P* < 0.05).

## Discussion

### Exogenous proline mitigated the toxic effects of Cd in *B. juncea*

In this study, Cd significantly inhibited the growth of *B. juncea*, while the application of exogenous proline increased the proline content (Fig. 1), protected the photosynthetic pigments (Fig. 3), and maintained photosynthesis (Fig. 4) and cell viability (Fig. 5), thus alleviating the inhibition of Cd on growth (Fig. 2). The results also showed that 60 mg/L was the optimal concentration of exogenous proline to alleviate the toxic effects of Cd in *B. juncea*. Similar results were observed in chickpea (*Cicer arietinum*) [23], olive plants (*Olea europaea* L. cv. Chemlali) [17], and wheat (*Triticum aestivum* L.) [12] under Cd stress, as well as arsenate-stressed eggplant (*Solanum melongena*) [24]. Interestingly, the alleviating effects of exogenous proline on cd toxicity in *B. jun*cea showed an approximate inverted U curve. Namely, 60 mg/L proline was most effective in alleviating cadmium toxicity, and the effect was weaker when it was lower or higher than 60 mg/L. The reason may be that excessive proline is detrimental to plants [25].

Cd stress can lead to the excessive accumulation of ROS, which will affect cell elongation and cell division, thus, hindering plant growth and development [12, 26]. Therefore, the inhibition of plant growth and development is considered as one of the vital indices to evaluate the toxic effects of Cd [24]. Proline plays multifarious roles, including adaptation, recovery, and signaling, when it comes to combating stress in plants [27]. The promotion of exogenous proline on plant growth and development may be related to its potential to stabilize subcellular compartments through the detoxification of free radicals and maintenance of the redox potential under Cd stress [12].

Previous studies have also reported substantial reductions in chlorophyll content and photosynthesis rates under Cd toxicity [28–30]. This could be explained by the effects on the synthesis of chloroplasts in cells and reduction in the content of chlorophyll, which hampers photosynthesis in Cd-stressed plants [31]. In our study, the addition of exogenous proline increased the content of chlorophyll in plant leaves. Similar studies showed that the addition of exogenous proline alleviated the loss of total chlorophyll and carotenoids in coriander under Hg stress [14]. Wani et al. [16] also reported that foliar spraying different concentrations of proline could improve the chlorophyll and photosynthetic characteristics in the two varieties of *B. juncea* (‘Varuna’ and ‘RH-30’). This can be explained by the ability of proline to protect the photosynthetic organelle of plants under abiotic stress [32]. The positive role of proline in protecting plants under stress is related to the electron transfer of proline between stable cell membranes and mitochondria [33].

Cd stress seriously affects the overall photosynthetic capacity of plants and destroys the photosynthetic machinery [34]. In this study, Cd inhibited the Pn and Gs of *B. juncea* leaves, while the Ci increased. The Ci levels at 2 d and 7 d were higher than those at 0 d (Fig. 4), which indicates that the decrease of Pn might be due to the non-stomatal limitation caused by the reduction in the fixation of CO_2_ by RuBisCo [35]. The Tr levels in the *B. juncea* leaves are reduced under Cd stress, possibly due to increased cellular stomatal resistance or even closure. This result is similar to the effect of Cd on the photosynthetic rate of hybrid *Pennisetum* [36] and mung bean [*Vigna radiata* (L.) Wilczek] seedlings [35]. The addition of exogenous proline mitigated the photosynthetic inhibition of leaves. This is similar to the studies of Cd on pigeon pea (*Cajanus cajan* L.) [37], and other stresses on chili (*Capsicum annuum*) [38] and sorghum (*Sorghum bicolor*) [39]. Stressed plants with exogenously added proline were more effective at assimilating CO_2_ compared to stressed plants, which may be associated with the increased levels of Gs and Tr. The enhancement of Tr was mainly due to the increase in Gs levels, which triggered enhanced CO_2_ diffusion within the leaf tissues [40]. The Ci increased through the enhancement of Gs, which, in turn, affected the water content of plant tissues under Cd stress. Therefore, exogenous proline may play an effective mechanism to mitigate photosynthesis inhibition in Cd-stressed *B. juncea*.

Exogenous proline reduced the Cd content in both the belowground part and the aboveground part of *B. juncea* exposed to Cd stress. In addition, the translocation coefficient of Cd content decreased following exogenous proline treatment (Fig. 6). This suggests that the translocation of Cd to the aboveground part was restricted, thus, protecting the photosynthetic apparatus of the leaves. Similar findings were reported in the application of exogenous proline in tobacco (*Nicotiana benthianum*) [41], olive [17], and date palm [18] under Cd stress. *In vitro* experiments showed that proline could form complexes with Cd [42]. Exogenous proline treatment also elevated the levels of GSH (Fig. 9) and PCs (Fig.S2), chelators of Cd. PCs form chelates with heavy metal ions in the cytosol and are subsequently transported into the vacuole [43]. It is worth considering that these chelators may play roles in the retention of Cd in the roots, thereby reducing the translocation of Cd to the aboveground part. Therefore, the application of exogenous proline can effectively limit the contents of Cd and alleviate the toxicity caused by Cd to *B. juncea*.

### Exogenous proline maintained the redox homeostasis in *B. juncea* under Cd stress

DAB and NBT staining showed that exogenous proline had a positive effect at ameliorating the accumulation of ROS in the response of *B. juncea* to Cd exposure, thus, protecting the plants from Cd toxicity (Fig. 7). A similar result was reported by Yu et al. [15], in which exogenous proline positively affected the amelioration of the lipid peroxidation of rice seedlings exposed to chromium.

Proline is an osmotic substance, which has an osmotic adjustment function and antioxidant activity [44]. It stabilizes the osmotic differences between the cell’s surroundings and cytoplasm, protects the plant cells from oxidative stress, and maintains the intracellular redox equilibrium [11, 45, 46]. Previous studies have shown that Cd stress can aggravate the degree of plasma membrane peroxidation, destroy the structure of the cell membrane, and lead to the accumulation of a large number of ROS in plants [30, 47, 48]. This can be explained by the direct or indirect involvement of proline in the process of ROS elimination, which may reduce the accumulation of ROS and the oxidative damage of Cd to the plasma membrane to some extent, thereby helping to maintain the normal physiological processes of *B. juncea*.

As shown in Fig. 8, the activities of antioxidant enzymes, including SOD, POD, APX, and CAT, in *B. juncea* seedlings leaves and roots treated with 60 mg/L proline were significantly higher than those of the control (*P* < 0.05). Thus, exogenous proline, particularly at a concentration of 60 mg/L, could improve the activities of these antioxidant enzymes in *B. juncea* seedlings under Cd stress. Xu et al. [49] had similar findings that proline could reduce the toxicity of Cd to black nightshade (*Solanum nigrum*) by detoxifying ROS and increasing the activities of antioxidant enzymes. In addition, Islam et al. [41] found that exogenous proline supplements could improve the activities of antioxidant enzymes, thus, enhancing the tolerance of tobacco to Cd stress. Heavy metal stress can impact the activities of some antioxidant enzymes, including SOD, POD, APX, and CAT in plants [50]. SOD is a defensive enzyme system that scavenges ROS, and the addition of exogenous proline could ensure that there is sufficient SOD to degrade H_2_O_2_ [33]. POD catalyzes the oxidation of several compounds using H_2_O_2_ as the electron acceptor [51]. APX acts a pivotal part in antioxidant defense by involving the fine regulation of ROS [52]. CAT catalyzes the conversion of H_2_O_2_ to water and molecular oxygen [50]. Therefore, the addition of exogenous proline may act as a protectant of antioxidative capacities to mitigate the toxicity of Cd.

In this study, the contents of GSH and GSSG in the leaves and roots of *B. juncea* under Cd stress increased, and the ratio of GSH/GSSG decreased. After the addition of exogenous proline, the GSH, GSSG, and GSH/GSSG ratios in the leaves and roots of *B. juncea* increased compared with the control (Fig. 9). GSH plays a critical role in the response of plants to oxidative stress [53, 54]. As an antioxidant, it can remove ROS, such as H_2_O_2_ and O_2_^−^ [55]. GSH has a high reducing ability and plays a multifunctional role in many biological processes, such as cell growth and division, sulfate transport, signal transduction, protein and nucleic acid synthesis, and the detoxification of exogenous drugs. In addition, the balance between GSH and GSSG actively maintains cellular redox homeostasis [56, 57]. It has been reported that GSH combines with Cd to reduce its toxicity [43]. The toxic effect of Hg on sea purslane (*Halimione portulacoides* L.) increased the content of GSH and decreased the ratio of GSH/GSSG [58]. Arsenate (As^V^), zinc, and nickel stress significantly reduced the GSH/GSSG ratio in rice [59] and seep monkeyflower (*Mimulus guttatus*) [60]. This could be owing to the ability of plant chelates derived from GSH to form complexes with heavy metal ions to alleviate heavy metal stress [13, 61]. When the plants were stressed, the GSH/GSSG ratio decreased owing to the consumption of GSH in the detoxification and metabolism of ROS, which led to a change of the redox state. Additionally, the GSH/GSSG signal activated various defense mechanisms in plants through the redox signal pathway [62].

In this study, Cd stress led to the accumulation of PCs in *B. juncea*. The induction of PCs reduces the damage caused by heavy metals by complexing excess heavy metals via thiol (-SH) and avoiding their intracellular circulation in the form of free ions [63]. Other studies also found that Cd stress caused a significant accumulation of PCs in plants [64–66]. Our study suggested that the addition of exogenous proline increased the GSH content, thereby increasing the synthesis of PCs, which improved the chelation of Cd and reduced the oxidative damage caused by Cd.

In the present experiment, the GR activity was enhanced by the addition of exogenous proline under Cd treatment (Fig. S3). Similarly, other studies have shown that the addition of exogenous proline significantly increased the level of GR under various environmental stresses [67–69]. GR uses NADPH as the electron donor to catalyze the reduction of GSSG to GSH [70], thus, playing a key role in maintaining the content of GSH and the redox state (GSSG/GSH) of the glutathione pool in plants [62]. Our results suggest that exogenous proline may maintain the GSH pool by enhancing GR activity, thereby maintaining higher GSH/GSSH ratios and consequently redox homeostasis.

Both GSH and proline belong to the *α*-Ketoglutarate family and synthesized from the same precursor glutamate [71]. Excess proline within plant cells can also be degraded to glutamate [72]. Therefore, it is possible that exogenous proline addition under Cd stress reduced the demand for endogenous proline synthesis, thereby reducing the consumption of L-glutamate for proline synthesis, which was beneficial for GSH synthesis. This may also be part of the reason why exogenous proline treatment increased the content of GSH. The relationship between proline metabolism and GSH metabolism under Cd stress needs further investigation.

## Conclusions

**Fig. 10:**
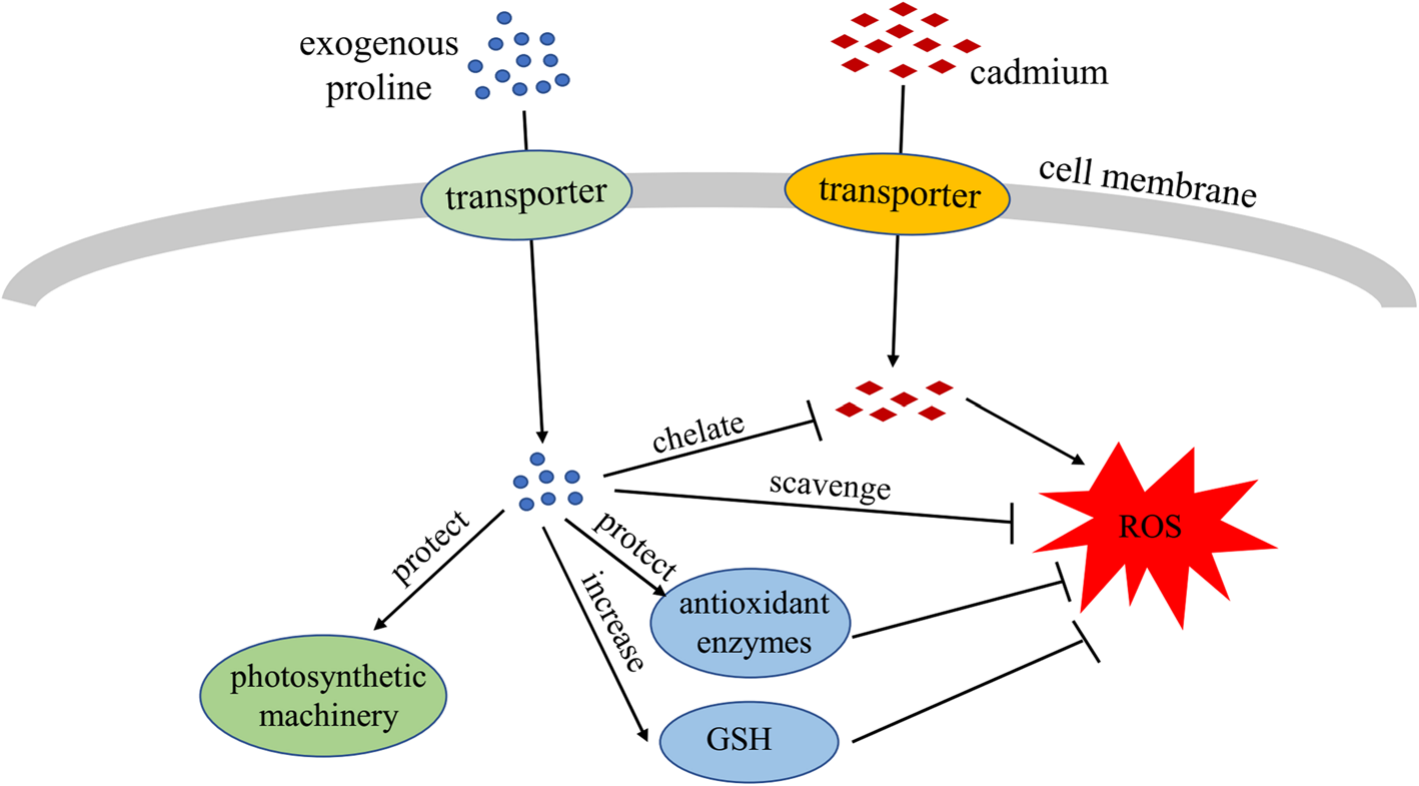
Overview of the potential mechanism of exogenous proline in alleviating cadmium toxicity in *Brassica juncea*

Exogenous proline protected the photosynthetic pigments, maintained photosynthesis and cell viability, and reduced the uptake and translocation of Cd, thus, alleviating the inhibition of Cd on growth in *B. juncea*. Exogenous proline treatment reestablished redox homeostasis by elevating the activities of antioxidant enzymes and nonenzymatic antioxidant contents, which, in turn, mitigated the toxicity of Cd to *B. juncea* (Fig. 10). Compared with other concentrations, 60 mg/L exogenous proline was the most effective at mitigating Cd toxicity in *B. juncea*. However, the molecular mechanism and related signaling pathway of proline in alleviating Cd toxicity in *B. juncea* merit further study.

## Materials and methods

### Plant growing conditions and treatment

*B. juncea* ‘Purple-leaf Mustard’, a rapeseed cultivar planted in Hunan, China, was kindly provided by Prof. Liu of Hunan Agricultural University [73] and preserved at the Central South University of Forestry and Technology. The seeds were sterilized with 70% ethanol and germinated for 2 days at 25 °C. The seedlings grown in one-half Hoagland nutrient solution were transferred to a climate chamber with a 14 h/10 h day/night alternate cycle, 200 µmol/m^− 2^s^− 1^ light intensity, and relative humidity of 55–75% at 25 °C.

After 40 days, seedlings of similar sizes were subjected to the following treatments: (1) Cd 80 µM + proline 0 mg/L (Control), (2) Cd 80 µM + proline 20 mg/L (T1), (3) Cd 80 µM + proline 40 mg/L (T2), (4) Cd 80 µM + proline 60 mg/L(T3), (5) Cd 80 µM + proline 80 mg/L (T4), and (6) Cd 80 µM + proline 100 mg/L (T5). In addition, to further compare the effects of control condition and the treatment of proline alone on plant growth and PCs content, and activities of GR, another four treatments were set up as follows: (1) normal growth (Control), (2) 80 µM Cd treatment (Cd), (3) 60 mg/L proline treatment (Pro), and 80 µM Cd + 60 mg/L proline treatment (Cd + Pro). Samples were taken at 0 d, 2 d, and 7 d for analysis. Previous studies have shown that 80 µM Cd in one-half Hoagland nutrient solution can cause significant toxic effects on *B. juncea* [74, 75]. The nutrient solution was changed every two days. Three replicates were established for each treatment.

### Detection of proline content

The contents of proline were determined as described by Bates et al. [76]. A total of 0.5 g of fresh leaves were ground in 5 mL of sulfosalicylic acid (3%, v/v) and then boiled for 10 min. The extract (2 mL) was added to 2 mL of acidic ninhydrin and boiled for 30 min. After cooling, 4 mL of toluene was added to the mixture and centrifuged at 3000 g for 5 min. The absorbance was measured at a wavelength of 520 nm with toluene as a blank. The proline content in the sample was calculated using the linear correlation equation of the standard curve of L-proline (Abs = 0.0406 C + 0.2327, *r*^2^ = 0.9825).

### Determination of plant height, root length, and dry weight

Plant height and root length were measured with a ruler. The fresh plants were placed in an oven at 65 °C and dried to a constant weight to measure the dry weight of shoots and roots.

### Measurement of chlorophyll and carotenoid content, net photosynthetic rate, stomatal conductance, intercellular carbon dioxide concentration, and transpiration rate

The contents of photosynthetic pigments were assayed as described by Petrovic and Krivokapic [77]. Fresh leaves (0.2 g) were ground in calcium carbonate (0.1 g) and 95% ethanol and measured the absorbances at 665 nm, 649 nm, and 470 nm wavelengths were measured using 95% ethanol as a blank.

$${\text{C}}_{\text{a}}\text{=13.95}{\text{A}}_{\text{665}}-\text{6.88}{\text{A}}_{\text{649}}$$

$${\text{C}}_{\text{b}}\text{=24.96}{\text{A}}_{\text{649}}-\text{7.32}{\text{A}}_{\text{665}}$$

$${C}_{x,c}=\frac{\text{1000}{\text{A}}_{\text{470}}-\text{2.05}{\text{C}}_{\text{a}}-\text{114.8}{\text{C}}_{\text{b}}}{\text{245}}$$

$$Content\ of\ chlorophyll\ pigment\ (mg/g)=\frac{{C * V * N}}{{m * 1000}}$$

*C*_*a*_: chlorophyll a; *C*_*b*_: chlorophyll b; *C*_*x,c*_: carotenoid content; *C*: pigment concentration (mg/L); *V*: volume of extract (mL); *N*: dilution ratio; *m*: sample mass (g).

The Pn, Gs, Ci, and Tr of *B. juncea* were measured by a portable photosynthetic system (LI-6400XT; LI-COR, Lincoln, NE, USA) with photosynthetic effective radiation of 100 µmol/m^− 2^s^− 1^, room temperature of 25 °C, and relative humidity of 70%.

### Analysis of cell viability

The cell viabilities of plant leaf and root were determined by the Evans blue staining method [78, 79]. The area of leaves that was dyed was calculated using Image J (NIH, Bethesda, MD, USA). The calculation formula is as follows:

$$V\left(Cell relative activity\right)=A\left(Non staining area\right)/{A}_{t}\left(Total leaf area\right)$$

### Determination of Cd content

The content of Cd was measured as described by Yang. et al. [47]. A total of 0.3 g of dry *B. juncea* leaf or root samples were ground into a powder digested with a diacid mixture of (HNO_3_/HClO_4_) (9:1, v/v), and then diluted to 50 mL with ultrapure water. The content of Cd was determined using a flame atomic absorption spectrophotometer (ICE3500; Thermo Scientific, Waltham, MA, USA) after filtration. The Cd standard curve (Abs = 0.46900 C + 0.019858, *R*^2^ = 0.997) was plotted using the Cd standard solution (China national standard sample No. GSB04-1721-2004) obtained from Guobiao (Beijing) Testing & Certification Co., Ltd. (Beijing, China).

### Histochemical detection of ROS

The presence of H_2_O_2_ and O_2_^−^ in leaves was detected by the DAB and NBT staining methods, respectively [80]. The leaves and roots were submerged in a solution of DAB or NBT and subjected to vacuum slightly, then shaken for 4 h under dark conditions before they were decolorized and photographed. In the presence of endogenous peroxidase, DAB polymerized at the sites of H_2_O_2_ accumulation, which formed a brown DAB polymer. The blue formazan precipitate produced by the reduction of NBT by O_2_^−^ became visible.

### Determination of antioxidant enzyme activities and MDA content

Fresh leaves (0.1 g) were homogenized in an ice bath with 1 mL of 0.1 M sodium phosphate buffer (pH 7.8). The homogenate was centrifuged at 8000 g for 10 min at 4 °C, and the supernatant was collected for subsequent analysis. The whole extraction procedure was conducted at 4 °C. The activities of the antioxidant enzymes (i.e., SOD, POD, APX, and CAT) were determined by kits purchased from Shanghai Sinobest Biotechnology Co., Ltd. (Shanghai, China). The content of MDA was assayed by the thiobarbituric acid (TBA) method [81].

### Measurements of contents of glutathione, glutathione disulfide, and phytochelatins

The contents of glutathione (GSH) and glutathione disulfide (GSSG) were measured as described by Griffith [82]. The fresh leaves and roots (0.5 g) were extracted with 2.5 mL of 5% sulfosalicylic acid and centrifuged at 12,000 g for 20 min at 4 °C. A volume of 50 µL of supernatant was mixed with 50 µL of 5% sulfosalicylic acid, 24 µL of 1.84 mol/L triethanolamine, and 50 µL of 10% vinylpyridine, and incubated at 25 °C for 1 h to remove the GSH. A volume of 706 µL of 50 mmol/L phosphate buffer (pH 7.5, containing 2.5 mmol/L EDTA), 20 µL 10mmol/L EDTA, 20 µL of 10 mmol/L NADPH, and 80 µL of 12.5 mmol/L 5,5’-dithio-bis(2-nitrobenzoic acid) (DTNB) were mixed and incubated at 25 °C for 10 min, and then 20 µL of 50 U/mL GR was added. A mixture of extracts without DTNB and phosphate buffer was used as a blank, and the absorbance at 412 nm at 3 min was measured. The content of GSSG in the plant tissue was calculated using a standard curve of GSSG.

The content of total glutathione (GSH + GSSG) was determined by replacing the vinyl pyridine with distilled water of equal volume. The content of GSH was determined by subtracting the content of GSSG from the total glutathione.

The content of phytochelatins (PCs) was measured as described by Keltjens and van Beusichem [83]. The contents of non-protein thiol (NPT) were first determined as described by Nagalakshmi and Prasad [84], and then the contents of PCs are calculated as follows:

$$P\left(content of PCs\right)=A\left(content of NPT\right)-B\left(content of GSH\right)$$

### Determination of glutathione reductase activities

The GR activities were determined as described by Foyer and Halliwell [85] with some modifications. Fresh leaves and roots (0.1 g) were homogenized in phosphate buffer solution (containing 1 mmol/L EDTA) and centrifuged at 12,000 g for 30 min at 4 °C. The supernatant was the enzyme extract. A volume of 1.35 mL of phosphate buffer (containing 1 mmol/L EDTA), 0.05 mL of 5 mmol/L GSSG solution, and 0.1 mL of enzyme solution were added to a centrifuge tube in sequence. Finally, 20 µL of 4 mmol/ L NADPH solution was added to initiate the enzymatic reaction. The absorbance values of the reaction mixture at 340 nm were recorded at 30 s intervals, starting 15 s after initiation and measured continuously to obtain data for at least six points. The decrease at 340 nm was read from 0.5 to 3.5 min to calculate the enzyme activity.

### Statistical analysis

The effects of treatments were compared with a one-way analysis of variance (ANOVA) using SPSS 26 (IBM, Inc., Armonk, NY, USA). The means were compared using Duncan’s multiple range test at *P* < 0.05. All the figures were produced using Origin 2018 (OriginLab, Northampton, MA, USA).

## Supplementary Information

**Additional file** **1:** **Fig. S1.** The plant height (A), root length (B), shoot dry weight (C), and root dry weight (D) of *Brassica juncea* under normal growth (Control), 80 µM Cd treatment (Cd), 60 mg/L proline treatment (Pro), and 80 µM Cd + 60 mg/L proline treatment (Cd + Pro). All the analyses were performed with three replicates. Error bars represent the standard deviations (SD). The different letters in the group indicate significantly different values between treatments (*P* < 0.05).

**Additional file 2:** **Fig. S2.** Effects of exogenous proline on contents of PCs in leaves (A) and roots (B) of *Brassica juncea* seedlings under normal growth (Control), 80 µM Cd treatment (Cd), 60 mg/L proline treatment (Pro), and 80 µM Cd + 60 mg/L proline treatment (Cd + Pro). All the analyses were performed with three replicates. Error bars represent the standard deviations (SD). The different letters in the group indicate significantly different values between treatments (*P* < 0.05). PCs, phytochelatins.

**Additional file 3:** **Fig. S3.** Effects of exogenous proline on GR activity in leaves (A) and roots (B) of *Brassica juncea* seedlings under Cd stress. All the analyses were performed with three replicates. Error bars represent the standard deviations (SD). The different letters in the group indicate significantly different values between treatments (*P* < 0.05). GR, glutathione reductase.

## Data Availability

The datasets used and/or analysed during the current study are available from the corresponding author on reasonable request.

## Abbreviations

Cd: cadmium
GSH: glutathione
GSSH: glutathione disulfide
ROS: reactive oxygen species
SOD: superoxide dismutase
POD: peroxidase
APX: ascorbate peroxidase
CAT: catalase
MDA: malondialdehyde
GR: glutathione reductase
Pn: net photosynthetic rate
Gs: stomatal conductance
Ci: intercellular carbon dioxide concentration
Tr: transpiration rate
DAB: 3,3′-diaminobenzidine
NBT: nitro blue tetrazolium
H_2_O_2_: hydrogen peroxide
O_2_^−^: superoxide anions
PCs: phytochelatins
DTNB: 5,5’-dithio-bis(2-nitrobenzoic acid)
